# Ghrelin inhibits autonomic response to gastric distension in rats by acting on vagal pathway

**DOI:** 10.1038/s41598-020-67053-y

**Published:** 2020-06-19

**Authors:** Mathieu Meleine, Lourdes Mounien, Karim Atmani, Wassila Ouelaa, Christine Bôle-Feysot, Charlène Guérin, Inge Depoortere, Guillaume Gourcerol

**Affiliations:** 1 Nutrition, Gut & Brain Unit (INSERM U1073), Institute for Biomedical Research and innovation, Rouen University, Rouen, France; 2 Université Clermont Auvergne, Inserm U1107, NeuroDol, Clermont-Ferrand, France; 3 Center for Cardiovascular and Nutrition Research, (UMR 1260 INRA/1263 INSERM), Aix-Marseille University, Marseille, France; 4 Gut Peptide Research Lab, Translational Research Center for GastroIntestinal Disorders, KU Leuven, Leuven, Belgium; 5 Department of Physiology, Rouen University Hospital, Rouen, France

**Keywords:** Sensory processing, Neurophysiology

## Abstract

Ghrelin is the only orexigenic peptide currently known and a potent prokinetic by promoting gastric motility but novel insights suggest that its role extends beyond satiety regulation. Whereas ghrelin was shown to provide somatic and colonic antinociception, its impact on gastric sensitivity is unknown even though stomach is a major ghrelin secreting tissue. Autonomic response to gastric mechanosensitivity was estimated by measuring blood pressure variation as a surrogate marker in response to gastric distension (GD) before and after ghrelin (or vehicle) administration. Involvement of spinal and vagal pathways in the ghrelin effect was studied by performing celiac ganglionectomy and subdiaphragmatic vagotomy respectively and by evaluating the expression of phosphorylated extracellular-regulated kinase 1/2 (p-ERK1/2) in dorsal root and nodose ganglia. Finally the phenotype of Ghrelin receptor expressing neurons within the nodose ganglia was determined by *in situ* hybridization and immunofluorescence. Ghrelin reduced blood pressure variation in response to GD except in vagotomized rats. Phosphorylated-ERK1/2 levels indicated that ghrelin reduced neuronal activation induced by GD in nodose ganglion. The effect of ghrelin on gastric mechanosensitivity was abolished by pre-treatment with antagonist [D-Lys^3^]-GHRP-6 (0.3 mg/kg i.v.). Immunofluorescence staining highlights the colocalization of Ghrelin receptor with ASIC3 and TRPV1 within gastric neurons of nodose ganglion. Ghrelin administration reduced autonomic response to gastric distension. This effect likely involved the Ghrelin receptor and vagal pathways.

## Introduction

Ghrelin is a 28-amino acid peptide produced by endocrine cells of the oxyntic gastric mucosa and, to a lesser extent, by hypothalamus, pituitary and the intestine^1,2^. This hormone exerts its action by binding the G protein-coupled ghrelin receptor expressed both in central nervous system and peripheral organs^2^. Acylation of the 3^rd^ serine confers to ghrelin an orexigenic activity that induces a subsequent increase in food intake following either central or peripheral administration^2^. Moreover ghrelin displays prokinetic properties by promoting propulsive activity of the stomach^2,3^. Taken into consideration the orexigenic and prokinetic actions of ghrelin, analogs have been recently tested to treat gastric-dysmotility disorders like in diabetic gastroparesis^4,5^.

For instance; the Ghrelin receptor agonist, relamorelin, has recently been studied in a phase 2 clinical trial enrolling diabetic gastroparesis patients^6^. Twelve weeks administration of 10, 30 or 100 µg of relamorelin resulted in a reduction of diabetic gastroparesis cardinal symptoms. However, symptomatic efficacy was not correlated with acceleration of gastric emptying induced by relamorelin. Concomitantly, several prokinetics failed to improve gastroparesis symptoms despite gastric emptying acceleration^7–9^. Those data suggest that gastric emptying acceleration is not a necessary condition for overall symptom improvement and the symptomatic efficacy of Ghrelin receptor agonist may imply additional mechanisms. Interestingly, in the aforementioned study, relamorelin reduced epigastric pain^6^. This may suggest that ghrelin pathway may be involved in visceral nociception. To date, although ghrelin has been shown to reduce somatic^10–13^ and colonic^14^ pain, its effect on gastric nociception remains uninvestigated whereas the stomach is a major ghrelin-producing site. Owing to the potent effect of ghrelin on gastric motility^3^, studying its impact on gastric mechanosensitivity in response to isobaric distension is challenging. This may explain why such effect has not been yet investigated. Therefore the aim of this study was to assess the effect of ghrelin on gastric mechanosensitivity and to determine the neuronal pathway potentially involved in its effects. This was achieved in atropinized rats, in order to prevent gastric pressure changes induced by ghrelin-stimulated gastric contractions.

## Results

### Ghrelin decreases the variation of BP in response to GD through activation of Ghrelin receptors

**Figure 1:**
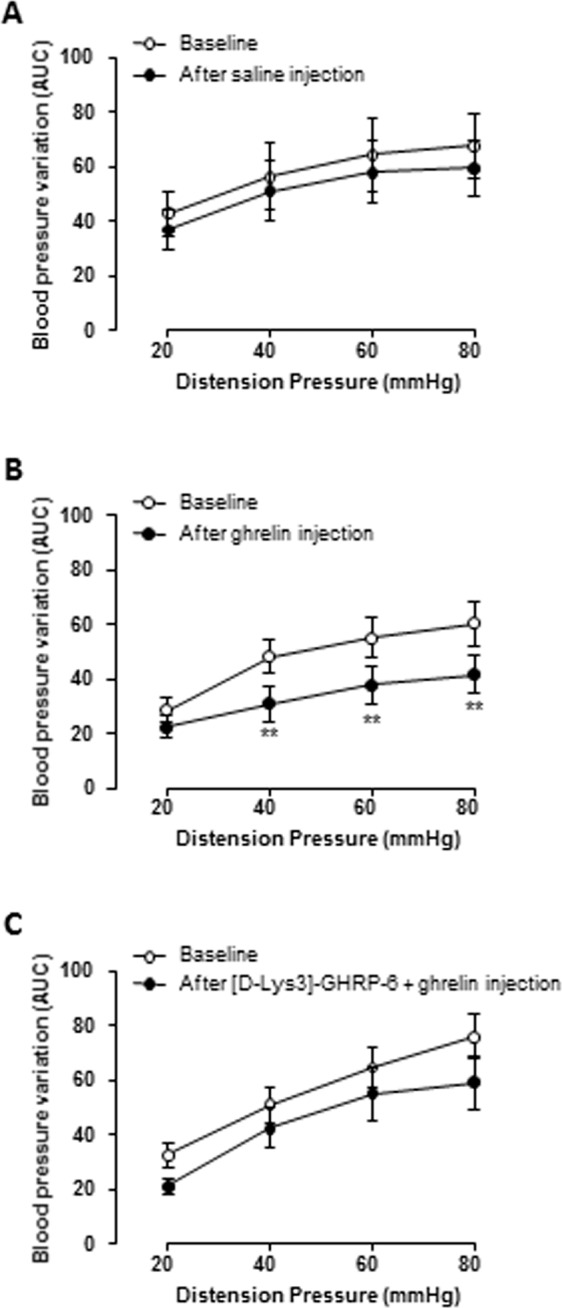
Effect of acylated ghrelin on the variation of blood pressure (BP). (**A**,**B**) Variation of BP in response to graded gastric distension (GD) of 20, 40, 60, 80 mmHg. A first set of GD (baseline) was followed by intravenous (iv) injection of vehicle (saline; n = 10, **A**) or acylated ghrelin at 30 µg/kg (n = 10, **B**), then a second set of GD was performed 5 minutes after injection. (**C**) Ten minutes before ghrelin administration the Ghrelin receptor antagonist [D-Lys^3^]-GHRP-6 was intravenously administered (n = 8). Data are expressed as mean ± SEM. **p < 0.01.

We first validated the influence of atropine in the anesthetized rat model on the variation of BP in response to GD. The variation of BP in response to graded GD was similar before and after i.v. atropine injection (1 mg/kg) followed by i.v. saline injection. (Fig. 1A). Using this model, we investigated the effect of acylated ghrelin on the variation of BP in response to GD. A decrease in the variation of BP was observed after i.v. ghrelin injection (30 µg/kg) compared to the saline injected group. This decrease yielded 34% at 40 mmHg (p < 0.01) and 60 mmHg of distension (p < 0.01) and 33% at 80 mmHg of distension (p < 0.01; Fig. 1B). By contrast, pretreatment with [D-Lys^3^]-GHRP-6 (0.3 mg/kg i.v.) abolished the effect of ghrelin on the variation of blood pressure in response to gastric distension (Fig. 1C). This effect appears to be specific to gastric stimulation and do not involve an alteration of vascular function since mean arterial pressure (MAP) monitored before the 2 distension sets and before and after ghrelin (or vehicle) intravenous administration did not differ between groups (Supp. Fig. 1).

**Figure 2:**
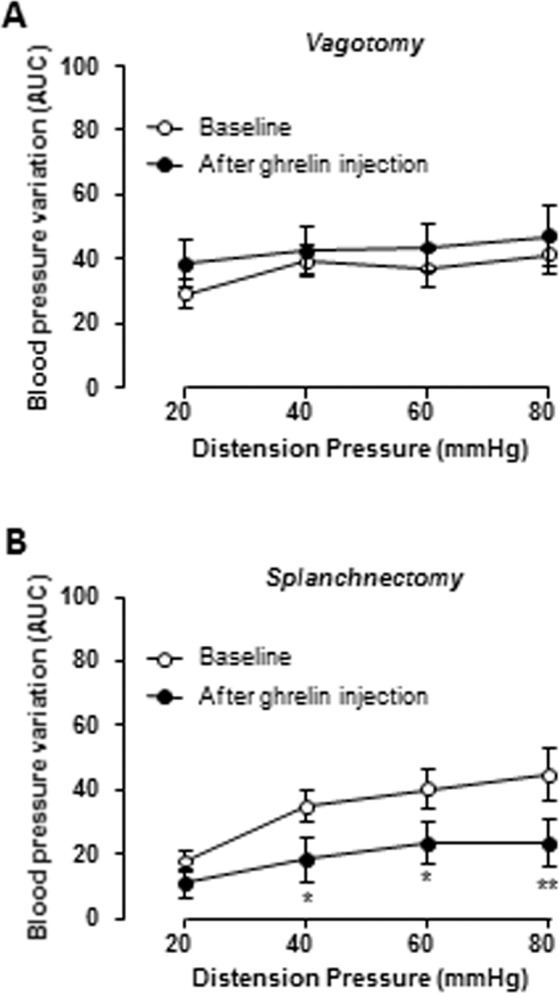
Effect of bilateral vagotomy and splanchnectomy on autonomic response to gastric distension following ghrelin administration. Variation of BP in response to graded gastric distension (GD) of 20, 40, 60, 80 mmHg. A first set of GD (baseline) was followed by intravenous (iv) injection of acylated ghrelin at (30 µg/kg i.v) in vagotomized (n = 10, A) and splanchnectomized (n = B) rats. Data are expressed as mean ± SEM.

### Effect of ghrelin on gastric mechanosensitivity involves preferentially vagal pathway

In this experiment we performed subdiaphragmatic vagotomy or celiac ganglionectomy in order to disrupt vagal and splanchnic innervation of the stomach respectively. The effect of ghrelin (30 µg/kg i.v.) was completely abolished in vagotomized rats (Fig. 2A) while it was preserved in rats with splanchnectomy (Reduction of 47% at 40 mmHg (p < 0.05), 42% at 60 mmHg (p < 0.05) and 48% at 80 mmHg (p < 0.01); Fig. 2B). It is of note that both vagotomy and splanchnetomy minimized the variation in blood pressure in response to gastric distension compared to naïve animals used in the first experiment (Supp. Fig. 2). This observation validates the effective surgical denervation of the stomach on gastric sensitivity. The effect of ghrelin in vagotomised rats unlikely involves an alteration in vascular function since mean arterial pressure measured before the 2 distension sets and before and after ghrelin administration is similar between groups (Supp. Fig. 1).

### Effect of the different treatments on gastric compliance

**Figure 3:**
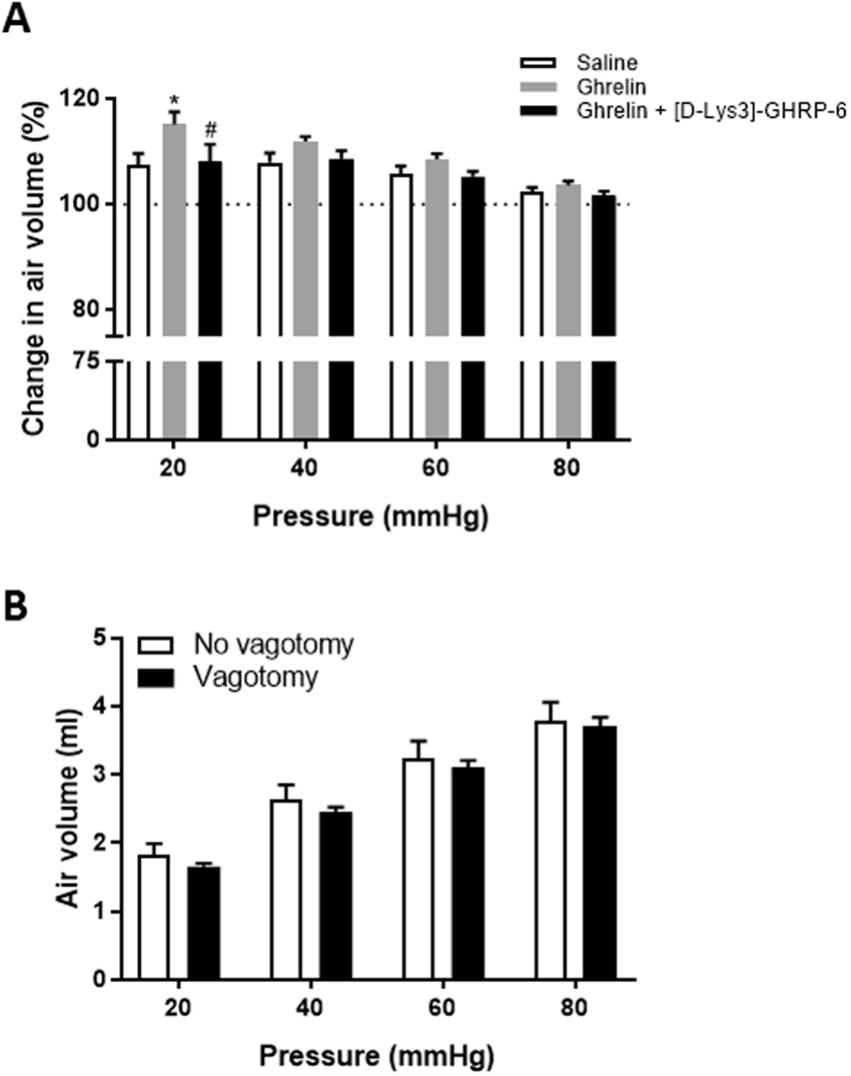
Effect of the different treatments and vagotomy on gastric compliance. (**A**) Percentage of change in intra-balloon air volume between the 2nd and the 1st distension sets.*p < 0,05 (vs. Saline); ^#^p < 0,05 (vs. Ghrelin). 2-way ANOVA (Time, Treatment) followed by Sidak’s multiple comparison post hoc test. (**B**) Intra-gastric balloon air volume corresponding to distension pressures in vagotomised and naïve rats.

In order to check the effect of ghrelin and [D-Lys^3^]-GHRP-6 on the intensity of gastric stimulation we compared gastric compliance between the 2^nd^ and the 1^st^ distension sets in saline, ghrelin and ghrelin + [D-Lys^3^]-GHRP-6 treated groups. Although ghrelin treatment significantly increased balloon volume during the 2^nd^ distension set for the 20 mmHg distending pressure compared to other groups, changes in air volume were similar for the others distending pressures suggesting that the intensity of gastric stimulation is not affected by the treatments (Fig. 3A). Gastric compliance was also compared between vagotomised and naïve rats for the 1^st^ distension set. No difference in intra-gastric balloon volume was observed between groups meaning that the surgery had no impact on the intensity of stimulation (Fig. 3B).

### Effect of ghrelin on neuronal activation

**Figure 4:**
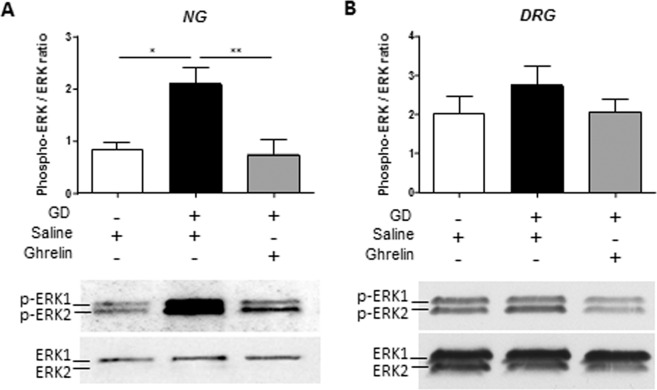
Effect of ghrelin on neuronal activation. Phosho ERK 1/2 on total ERK1/2 ratio was assessed by Western Blot in sham (n = 8) and distended rats previously treated with saline (n = 8) or ghrelin (30 µg/kg i.v., n = 12).

Repeated nociceptive gastric distensions increased p-ERK1/2/ERK1/2 ratio compared to non-distended rats in nodose ganglion (2.09 ± 0.15 vs. 0.86 ± 0.30, p < 0.05) but not in T8-T9 DRGs (Fig. 4A,B). Ghrelin administration (30 µg/kg i.v.) restored p-ERK1/2/ERK1/2 ratio to basal level in nodose ganglion compared to rats treated with vehicle (0.74 ± 0.28 vs. 2.09 ± 0.15, p < 0.01).

### Expression and phenotype of Ghrelin receptors in NG

**Figure 5:**
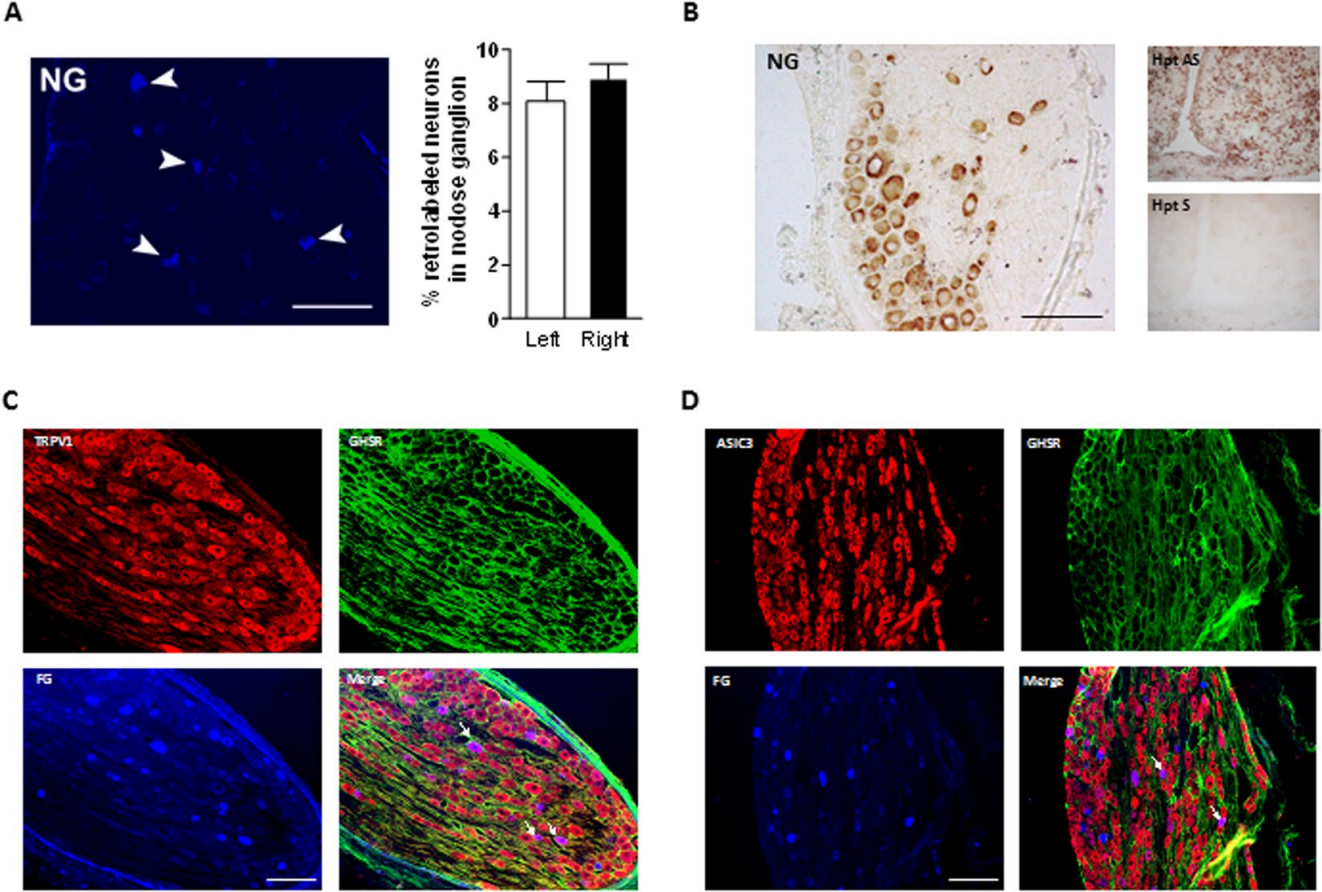
Expression and phenotype of gastric Ghrelin receptor neurons in nodose ganglia. (**A**) Gastric neurons were identified by Fluorogold retrotracer injection and percentage on gastric neurons was assessed in left and right nodose ganglia. (**B**) *In situ* hybridization against *ghsr1a* mRNA was performed in nodose ganglia (left panel). Test of the antisense probe (upper right panel) and sense probe (lower right panel) in the hypothalamus. (**C**) Retrolabeled gastric neurons were stained for Ghrelin receptor and TRPV1 by immunofluorescence. (**D**) Retrolabeled gastric neurons were stained for Ghrelin receptor and ASIC3 by immunofluorescence. Scale bar = 200 µm.

Eight to 9% of afferent neurons in nodose ganglia were stained following Fluorogold retrotracer injection in the stomach. No difference was observed in the numbers of retrolabeled neurons between left and right ganglia (Fig. 5A) therefore no distinction was made between left and right side in the following *in situ* hybridization and immunofluorescence experiments. *In situ* hybridization showed a strong expression of Ghrelin receptor mRNA nodose ganglion that was not observed when slices were incubated with the sense probe (Fig. 5B). Immunofluorescence staining displayed expression of Ghrelin receptor only at the plasma membrane in a majority of neurons from nodose ganglia (Fig. 5C,D). Of these a subset of neurons expressed also TRPV1 (Fig. 5C) or ASIC3 (Fig. 5D) channels.

**Figure 6:**
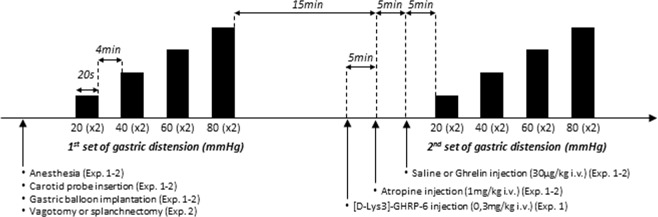
Gastric distension and administration protocol. (**A**) After anesthesia, a carotid pressure transducer and an intragastric balloon were implanted in rat. A first set of gastric distension ranging from 20 to 80 mmHg with 20 mmHg increments and 4 min resting intervals between distensions was performed. Each distension lasted 20 s and was repeated twice. After 15 min recovery atropine (1 mg/kg i.v.) was administered then saline or ghrelin (30 µg/kg i.v.). Five minutes after the injection a second set of gastric distension was performed. In the third experiment the Ghrelin receptor antagonist of [D-Lys3]-GHRP-6 (0.3 µg kg i.v.) was injected 10 minutes before ghrelin administration.

## Discussion

In the present report, we showed that acylated ghrelin decreased the variation of BP in response to GD, without change in gastric compliance, in anesthetized rats. This effect was prevented by pre-treatment with the Ghrelin receptor antagonist [D-Lys^3^]-GHRP-6. Reduction of the autonomic response to GD following ghrelin systemic administration involved a vagal pathway as demonstrated by the lack of effect observed in vagotomized rats and the decrease in neuronal activation, estimated by phosphorylated ERK1/2 expression, in nodose ganglion of rats treated with ghrelin. In this structure a subset of gastric neurons expressing Ghrelin receptors also expressed ASIC3 or TRPV1 ion channels that have previously been shown as key modulators of gastric mechanosensitivity^15^. Taken together, our data support a role for acylated ghrelin in the modulation of gastric mechanosensitivity to distension through the involvement of Ghrelin receptor and vagal afferents.

The antinociceptive or antihypersensitive action of ghrelin was previously reported in somatic pain^10–13^ and colonic distension models^14,16^. In the stomach, the role of ghrelin on gastroesophageal vagal afferents excitability has already been investigated on mouse *ex vivo* preparation by decreasing the response of tension receptors^17^. This inhibition involved Ghrelin receptor since it was reversed by [D-Lys^3^]-GHRP-6. In line with this study our results show a reduction of BP variation, used as a surrogate marker of gastric sensitivity in response to graded distensions, after ghrelin administration. Assessment of gastric sensitivity *in vivo* remains challenging since accessibility of the stomach for balloon insertion requires invasive surgical technique and nociceptive response necessitate viscero-somatic^18^ or viscero-visceral^19^ reflex recordings either in awake or anesthetized animals. We chose to work on a model anesthetized with thiobutabarbital because of its minimal impact on cardiovascular system and it provides a sufficient long-lasting anesthesia to perform the whole procedure^20^. In response to gastric distension we recorded changes in BP as a viscero-visceral reflex. This cardiovascular reflex implies a complex interplay between parasympathetic vagal and sympathetic splanchnic fibers. A recent report established a prominent role for vagal afferents in the trigger of the cardiovascular reflex using low pressure distension whereas splanchnic nerves are preferentially involved during high pressure distension^19^. Our data indicate a more pronounced effect of ghrelin at 40, 60 and 80 mmHg. Therefore, pharmacological action of ghrelin was expected to recruit splanchnic rather than vagal afferent. We demonstrated that intact vagal innervation is necessary for ghrelin to exert its action and the peptide prevents vagal neuronal activation induced by gastric distension in rats. Nevertheless the aforementioned study determined that an intact vagus nerve was essential for the complete eliciting of the cardiovascular reflex even during high pressure distension. This finding suggests a necessary crosstalk between vagal and splanchnic afferents to modulate cardiovascular responses to gastric distension with high intensity and ghrelin, by primary acting on vagal inputs, could modulate splanchnic regulation of the reflex. On the other hand, despite the effect of ghrelin was still observed in splanchnectomized rats, we cannot rule out its potential effect on spinal afferents. Indeed, Ghrelin receptor has been shown to be expressed in embryonic chick DRG^21^ and unpublished data obtained in our laboratory demonstrated that it is also expressed within T9-T10 DRG in rats. Moreover a recent report investing the effect of ghrelin in a rat model of colonic hypersensitivity pointed out an inhibited expression of TRPV1 channel in DRG neurons after ghrelin treatment^22^. Our data did not demonstrate such involvement of splanchnic pathway since we did not observe any effect of gastric distension on neuronal activation in DRG neurons and, as a consequence, no modulation by ghrelin administration. This lack of effect of gastric distension may be explained by the methodology we used. Compared to a previous study that demonstrated increased expression of p-ERK1/2 in retro-labeled gastric DRG neurons following gastric distensions by immunofluorescence^22^, we chose western blotting analysis. Since DRG afferent neurons innervate not only viscera but also somatic and articular territories, the signal could have been drowned into non-specific afferent messages.

The inhibitory effect of ghrelin observed in our work clearly involved Ghrelin receptor since pre-treatment with the antagonist [D-Lys^3^]-GHRP-6 prevented this effect. The involvement of Ghrelin receptor in the effect of acylated ghrelin has been clearly established in gastric motility or food intake^23,24^. However, its specific involvement in the sensitive action of ghrelin remains debated. Indeed, divergent observations have been made regarding the effect of ghrelin on somatic sensitivity. For instance, Erriquez *et al*., show that ghrelin or [D-Lys^3^]-GHRP-6 administered separately induced a change in cytosolic calcium concentration in glia and neurons of embryonic chick DRG^21^. On the other hand, Sibilia *et al*., found that [D-Lys^3^]-GHRP-6 failed to prevent the anti-hyperalgesic and anti-inflammatory effects exerted by ghrelin, thus suggesting that Ghrelin receptor was not involved in ghrelin action. In addition, administration of desacyl-ghrelin, which does not bind Ghrelin receptor, induced a significant reduction of the hyperalgesic and edematous response to inflammatory pain^12^. Our data are consistent with prior observations made on mechanically stimulated vagal afferents in which administration of [D-Lys^3^]-GHRP-6 abolished the decrease of tension receptors response induced by ghrelin^17^. To date, whether ghrelin acts through a paracrine or an endocrine pathway to modulate GD-sensitive afferent fibers is not known. In the present work, we used higher dose of acylated ghrelin compared to the level of physiological circulating levels. In fact, the half-life of acylated ghrelin in the blood is relatively short (<30 min) since acylated ghrelin is degraded by plasma esterase^25^. Ghrelin receptor has been found in nerve afferent endings in the stomach where receptors are transported to the periphery^26,27^. Moreover, the blood to brain transport of acylated ghrelin appears to be highly regulated^28^. Therefore, the concentration of acylated ghrelin is likely to be much higher at the periphery where afferent fibers would be an obvious site of action.

As suggested by immunofluorescence analysis, Ghrelin receptor colocalizes with TRPV1 or ASIC3 channels within gastric afferents of the nodose ganglion. Although these data do not allow us to conclude about the mechanism of action of ghrelin in vagal afferents it indicates that the ghrelin receptor is expressed in sensory fibers that also express key modulators of gastric mechanosensitivity. Indeed, vagal afferents from mice lacking ASIC3 and TRPV1 display blunted responses to distension on *in vitro* preparation compared to wild-type controls with a more pronounced effect of TRPV1 deletion^15^. It was previously shown that Ghrelin receptor can modulate the activity of ion channels and particularly of voltage-gated calcium channels Cav2.1 and Cav2.2 in rat and mouse hypothalamic neurons either by reducing the channel density at the membrane via the constitutive activity of the ghrelin receptor or by altering Cav2 gating via the binding of ghrelin to the receptor^29^. However, further experiments, using knock out strategies are warranted to assess the potential interplay between Ghrelin receptor and TRPV1 or ASIC3. In this particular case, pharmacological strategies blocking either TRPV1 or ASIC3 channels may not be relevant since it would result in an overall decreased gastric mechanosensation and thus would not allow to observe antinociceptive effect of ghrelin.

The therapeutic interest of ghrelin has been highlighted by numerous animals and human studies with beneficial effects in gastroparesis and postoperative ileus by promoting gastric emptying (for review)^30^. Interestingly ghrelin and others Ghrelin receptor agonists have also been shown to relieve nausea and vomiting in animal models and patients under chemotherapy^31^ or with diabetic gastroparesis^32^. Although Ghrelin receptor agonists appear to be a new promising pharmacological target to treat nausea and vomiting, the effect of a chronic administration on symptoms remains unknown. As described earlier, Ghrelin receptor agonist like relamorelin alleviates gastroparetic symptoms and accelerates gastric emptying but not necessarily in a concomitant fashion. On the other hand, visceral hypersensitivity is associated with functional dyspepsia, which shares clinical features with gastroparesis, including epigastric pain. It can therefore be speculated that Ghrelin receptor agonists acts on gastric nociceptive pathways, although this remains to be investigated in patients. Therefore, and in accordance with this previous report, our data demonstrate that ghrelin, by acting on Ghrelin receptor on vagal afferents could modulate gastric sensitivity and may be a therapeutic option of interest to treat gastroparetic patients with visceral hypersensitivity.

## Conclusion

We showed in the present work the ability of acylated ghrelin to decrease the autonomic response to gastric distension likely through the Ghrelin receptors and vagal afferents. This offers thus a new therapeutic target to treat symptoms related to the alteration of gastric sensory processing that includes nausea, vomiting, or pain. Further work both on animal models and in patients is therefore warranted to further explore the role of Ghrelin receptor in visceral pain processing.

## Methods

### Animals

Male Sprague-Dawley rats (350–450 g; Janvier, Le Genest-St-Isle, France) were housed in an animal facility that was maintained at 22 °C with an automatic 12-hour light/dark cycle. Animals had free access to standard chow (RM1 diet; SDS, Witham, Essex, UK) and drinking water. All experiments were performed in anaesthetized animals using sodium thiobutabarbital (Inactin ®, Sigma, Steinheim, Germany) at a dose of 200 mg/kg, given intraperitoneally (i.p.). Animal care and experimentation complied with both French and European community regulations (Official Journal of the European Community L 358, 18/12/1986). Experiments were approved by the National ethical committee for animal experimentation (CENOMEXA) and the French Ministry of Higher Education and Research (Ethical agreement No. 1008–01).

### Gastric sensitivity measurement in anesthetized rats

Gastric sensitivity was assessed using the variation of the arterial blood pressure (BP) in response to GD^33^. The BP was measured continuously in anesthetized animals using a perfused catheter (NaCl 0.9%; heparin 0.3%) introduced into the left carotid and connected to a pressure transducer (Millar Instruments, Houston, TX, USA). Pressure signal was recorded using Spike2 software (Cambridge Electronic Design Limited, Cambridge, United Kingdom) and processed by applying a DC remove (time constant: 20 s) and a smoothing (time constant: 5 s). Blood pressure variation was quantified by measuring the modulus of the processed signal during the 20 s of each distension.

### Gastric distension in rats

A spherical infinitely compliant distension balloon (diameter: 3 cm; maximum volume 12 mL in rats) was made using a polyethylene bag attached to a tube in polyethylene (Dutscher, Brumath, France) drilled in its extremity. The balloon was inserted in non-fasted anaesthetized rats through an incision at tip of the proximal stomach. Prior to balloon insertion, the gastric content was removed by gentle suction/reflux of warmed (32 °C) saline solution in order to mix gastric content until the stomach was emptied. The balloon was then connected to an electronic barostat (G&J Electronics Inc, Toronto, Canada) to perform isobaric graded GD. Distension paradigm consisted in balloon inflation from 20 to 80 mmHg by 20 mmHg increments in rats. Each distension lasted 20 seconds, was repeated twice and separated by a 4 min interval from the next (Fig. 6)^20,34^.

### Experimental protocols

#### Experiment 1: Effect of acylated ghrelin on variation of BP to graded GD in anesthetized rats

In anesthetized rats, a first set of graded GD was performed. Fifteen minutes later, atropine (1 mg/kg) was injected intravenously (i.v.) to prevent acylated ghrelin stimulation of gastric motility that would have jeopardized isobaric distension of the stomach. Five minutes after atropine injection, acylated ghrelin (30 µg/kg; Phoenix Pharmaceuticals, Burlingame, CA) or saline was injected i.v. (n = 10/group). A second series of GD was started 5 min later. In one experiment, [D-Lys^3^]-GHRP-6 (Phoenix Pharmaceuticals, Burlingame, CA), a Ghrelin receptor antagonist, was injected (0.3 mg/kg i.v.) 10 min before acylated ghrelin injection (n = 8). The dose of peptides were selected accordingly to our previous report as the maximal effective dose to accelerate gastric emptying^3^ (Fig. 6).

#### Experiment 2: Characterization of the neural pathway involved in the effect of acylated ghrelin on gastric mechanosensitivity

In a second experiment vagal or spinal denervation of the stomach was performed prior to the first set of gastric distension by subdiaphragmatic vagotomy (n = 10) or celiac ganglionectomy (n = 6), respectively. Then the same protocol as described in experiment 1 was performed (Fig. 6).

#### Experiment 3: Effect of acylated ghrelin on neuronal activation

In a third set of experiments, ERK1/2 phosphorylation was assessed by western blotting (see protocol below) in nodose ganglion (NG) and dorsal root ganglia (DRGs) 5 min after GD composed of 10 successive phasic distensions at 60 mmHg (20 s) separated by 40 s intervals following acylated ghrelin (n = 12) or vehicle (n = 8) administration, or in the absence of GD (balloon inserted but not inflated; n = 8).

### Western blotting analysis

Nodose ganglia, T8 and T9 DRGs from rats were homogenized in ice-cold lysis buffer containing 0.1% protease inhibitor cocktail (Sigma-Aldrich, Saint Quentin Fallavier, France). Vials were placed on ice for 15 min and then centrifuged for 15 min at 4 °C and 12,000r.p.m. The supernatant containing proteins was collected and stored at −80 °C until analysis. Proteins (25 μg) were separated on 4–12% Tris-glycine resolving gels (Invitrogen, Cergy-Pontoise, France). Proteins were then transferred to a nitrocellulose membrane (GE Healthcare, Orsay, France), which was blocked for 1 h at room temperature with 5% (w/v) non-fat dry milk in Tris buffered saline (TBS) (10 mmol/l Tris, pH 8; 150 mmol/l NaCl) plus 0.05% (w/v) Tween 20. Then, an overnight incubation at 4 °C was done with rabbit anti-phospho ERK1/2 antibody (1:2000; Cell signaling, Leiden, The Netherlands). After three washes in a blocking solution of 5% (w/v) non-fat dry milk in TBS/0.05% Tween 20, 1 h incubation with peroxidase-conjugated goat anti-rabbit IgG (1:5,000; Santa Cruz Biotechnology, Tebu-bio, Le Perray en Yvelines, France) was performed. After three additional washes, immunocomplexes were revealed by using the ECL detection system (GE Healthcare). Membrane was then stripped and the same protocol was performed using rabbit anti-ERK1/2 antibody (1/1000, Cell Signaling, Leiden, The Netherlands). Protein bands were quantified by densitometry using ImageScanner III and ImageQuant TL software (GE Healthcare) and phospho-ERK1/2 / ERK1/2 ratio was calculated to estimate neuronal activation.

### *In situ* hybridization

Total RNA from hypothalamus was reverse transcribed to generate a 559-bp DNA fragment of Ghrelin receptor. The reverse transcription product was amplified by PCR using the following primers: forward primer, 5′-TGTGGTGGTGTTTGCTTTCATCC-3′; and reverse primer, 5′-CCTGCTGTGGGTATGAGTTGT-3′ (IDT). The amplified 559-bp PCR product was subcloned into the pGEM-T vector, and the resulting plasmid was linearized and transcribed with T7 or SP6 RNA polymerase to generate antisense or sense probes, respectively. The probes were labeled by incorporation of digoxigenin (Dig)-11-UTP (Roche Diagnostics, Meylan, France).

Frozen sections (10-μm thick) were cut through the entire length of the NG. All tissue sections were mounted on gelatine-coated slides and stored at −80 °C. Single *in situ* hybridization with Ghrelin receptor probes was performed as previously described^35^. Briefly, sections were initially fixed in 4% paraformaldehyde, acetylated, treated with Triton X-100 (0.2%) and covered with prehybridization buffer (50% formamide, 0.6 M NaCl, 10 mM Tris-HCl, pH 7.5, 0.02% Ficoll, 0.02% polyvinylpyrollidone, 0.1% bovine serum albumin, 1 mM EDTA, pH 8.0, 550 μg/ml denatured salmon sperm DNA, 50 μg/ml yeast tRNA). Hybridization was performed overnight at 55 °C in the same buffer (except for salmon sperm DNA, the concentration of which was lowered to 60 μg/ml) supplemented with 10 mM dithiothreitol, 10% dextran sulfate and Dig-labeled probes (1%, v/v). Sections were washed in 2 × SSC at 60 °C and treated with RNase A (50 μg/ml) at 37 °C for 1 hour. The, sections were washed in a Tris-NaCl buffer (0.15 M NaCl, 0.1 M Tris-HCl, pH 7.5; buffer 1) and incubated for 30 minutes in a buffer containing 2% blocking agent (Roche Diagnostics) in NaCl/maleic acid buffer (0.15 M NaCl, 0.1 M maleic acid, pH 7.5). The sections were incubated overnight at 4 °C in buffer 1 containing anti-Dig Fab fragments conjugated to alkaline phosphatase (Roche Diagnostics) diluted 1:200 and 1% normal fetal bovine serum. The sections were rinsed in buffer 1 for 10 minutes and in a 0.1 M Tris-HCl buffer supplemented with 50 mM MgCl2 and 0.1 M NaCl, pH 9.5, for 10 minutes. The sections were then incubated in the chromogen solution containing 4-nitroblue tetrazolium-chloride and 5-bromo-4-chloro-3-indolyl phosphate supplemented with levamisole (2.4 mg/10 ml) for 2 hours. The reaction was stopped by two successive washes in a 10-mM Tris-HCl buffer, pH 8.0, containing 1 mM EDTA. NG sections were mounted with Glycergel (Dako, Trappes, France).

### Gastric neurons retro labeling

Laparotomy was performed under Ketamine (10mg/kg)/Xylazine (75 mg/kg) anesthesia in rats (n = 3). Ten to 12 injections of 0.5 to 1.5 µL of Fluorogold 4% in NaCl 0.9% equally distributed between fundus and antrum were realized and injection sites were thoroughly rinsed with warm NaCl 0.9% to prevent retrotracer spreading to adjacent structures. Muscular layer was closed with 6-0 absorbable sutures and the skin with 4-0 non-absorbable sutures (Ethicon). After 7 days recovery rats were terminally anesthetized with intraperitoneal pentobarbital injection and transcardially perfused with 75 mL warm NaCl 0.9% then 500 mL paraformaldehyde 4% in PBS. Left and right nodose ganglia were harvested, post-fixed overnight at 4 °C in 4% PFA and 10 µm sections were performed using a cryostat before mounting on slides.

### Immunohistochemistry

Simultaneous detection of Ghrelin receptor and ASIC3 or TRPV1 was performed in retrolabeled NG. 10-μm frontal sections were preincubated for 1 h with 0.1 M PBS (pH 7.4) containing 0.3% Triton X-100, and 5% fetal bovine serum (FBS), then incubated overnight at 4 °C with mouse monoclonal anti-Ghrelin receptor antibody (1:200, SC-374515, Santa Cruz Biotechnology, Germany), and guinea pig polyclonal anti-ASIC3 antibody (1:250, AB5927, Millipore, USA) or rabbit polyclonal anti-TRPV1 antibody (1:200, ACC-030, Alomone Labs, Israel) diluted in the same buffer. After washing, the sections were incubated for 90 min with the secondary antibody conjugated either to Cy-3 or Alexa Fluor 488 (1:500; all secondary antibodies were purchased from Life Technologies). In all conditions, sections were finally coverslipped with mounting medium for fluorescence microscope preparation. Sections were observed with Zeiss Axio Imager Z1 microscope equipped with AxioCam Icc3 digital camera (Carl Zeiss, S.A., Barcelona, Spain). Appropriate positive controls were used to check antibody specificity.

### Statistical analysis

Data are expressed as mean ± SEM. For gastric sensitivity experiments, a 2-way ANOVA (Treatment; Distension pressure) was used to compare the variation of BP between the first and second set of distension. For neuronal activation experiments, a Shapiro-Wilk test was performed to assess samples distribution. If distribution was normal in all the groups, differences among groups were assessed using one-way ANOVA followed by a Tukey’s or Sidak’s multiple comparison test. If one of the group had a non-normal distribution, a non-parametric Kruskal-Wallis followed by a Dunn’s multiple comparison test were performed. A p-value of <0.05 was considered statistically significant.

## Supplementary information

Supplementary Information 1.

Supplementary information 2.

## Data Availability

The data that support the findings of this study are available from the corresponding author, upon request.
