# Supplementary material for: Ghrelin inhibits autonomic response to gastric distension in rats by acting on vagal pathway
Source: Sci Rep. 2020 Jun 19;10:9986. doi: 10.1038/s41598-020-67053-y (PMC7305309; doi:10.1038/s41598-020-67053-y)
Supplement: Supplementary file 1 — Supplementary Information 1. [file 41598_2020_67053_MOESM1_ESM.docx]

**Ghrelin inhibits autonomic response to gastric distension in rats by acting on vagal pathway.**

MELEINE Mathieu, MOUNIEN Lourdes, ATMANI Karim, OUELAA Wassila, BÔLE-FEYSOT Christine, GUERIN Charlène, DEPOORTERE Inge, GOURCEROL Guillaume.

**Supplementary Information**


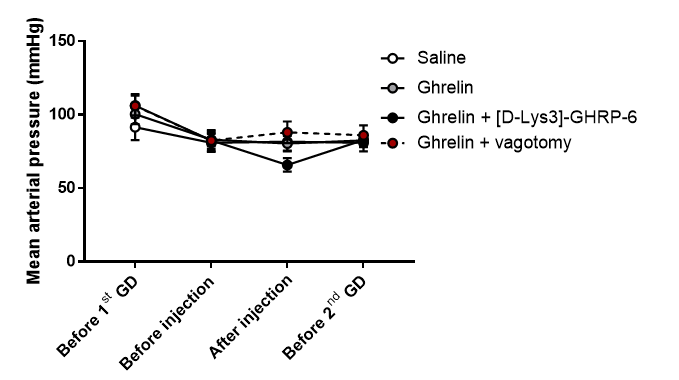


**Supplementary figure 1 : Effect of the different treatments on Mean Arterial Pressure (MAP)**. Mean arterial pressure was recorded during 1 minute just before the 1^st^ and 2^nd^ distension sets and during 2 minutes before and after ghrelin injection in each group. 2-way ANOVA (Time, Treatment) followed by Tukey’s multiple comparison post hoc test. GD: gastric distension.


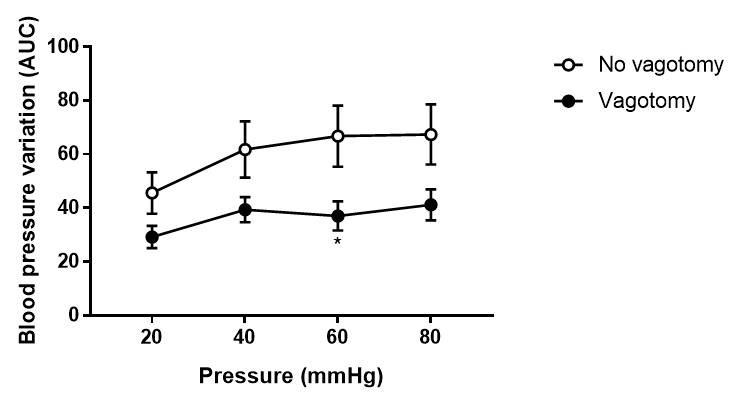


**Supplementary figure 2 : Effect of vagotomy on Mean Arterial Pressure (MAP) to gastric distension**. Blood pressure variation in response to gastric distension in vagotomised and naive rats * p<0,05. 2-way ANOVA (Pressure, Surgery) followed by Sidak’s multiple comparison post hoc test.
