# Supplementary material for: Ghrelin inhibits autonomic response to gastric distension in rats by acting on vagal pathway
Source: Sci Rep. 2020 Jun 19;10:9986. doi: 10.1038/s41598-020-67053-y (PMC7305309; doi:10.1038/s41598-020-67053-y)
Supplement: Supplementary file 2 — Supplementary information 2. [file 41598_2020_67053_MOESM2_ESM.pdf]

Ghrelin inhibits autonomic response to gastric distension in rats by acting on vagal pathway.

MELEINE Mathieu, MOUNIEN Lourdes, ATMANI Karim, OUELAA Wassila, BÔLE-FEYSOT Christine,
GUERIN Charlène, DEPOORTERE Inge, GOURCEROL Guillaume

Supplementary Info: Original scans of Western Blots

Nodose Ganglia
p-ERK

Experiment 1

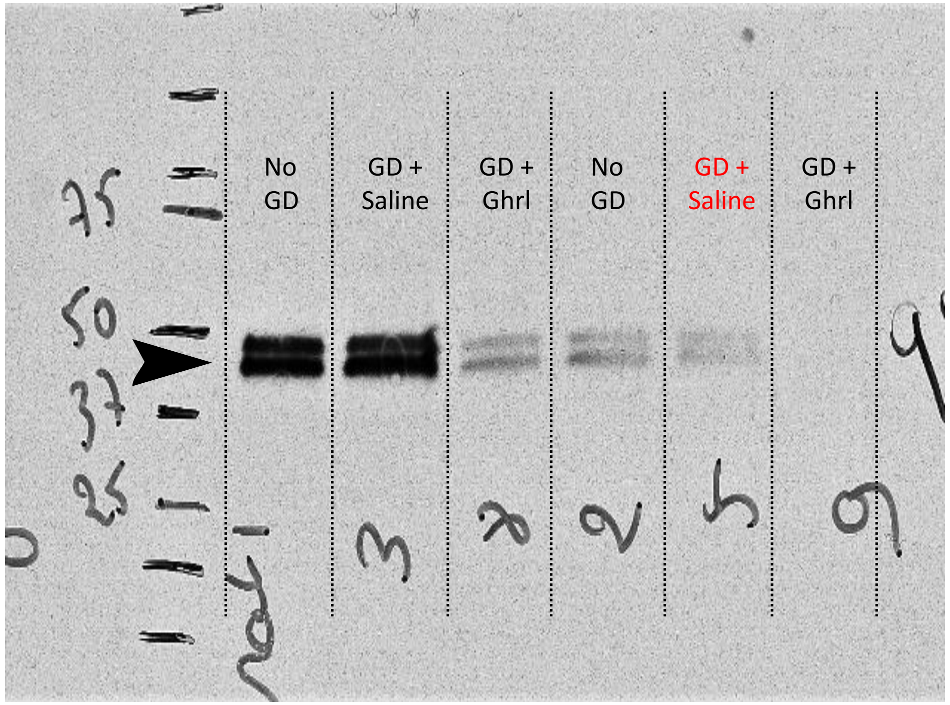

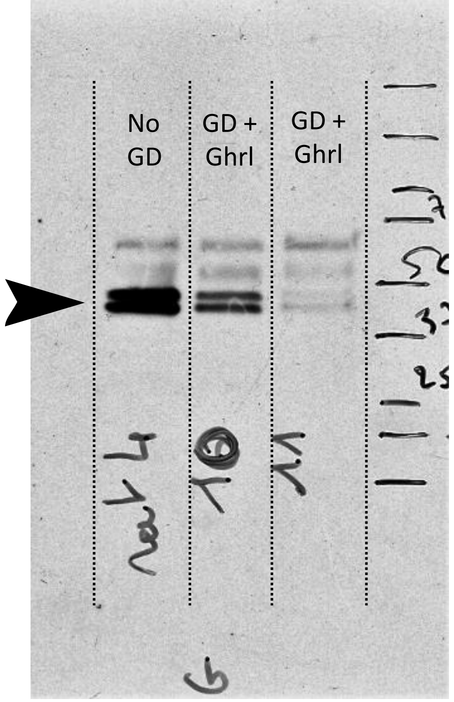

Data excluded because of a leak in gastric balloon to the small intestine

Nodose Ganglia
Total ERK

Experiment 1

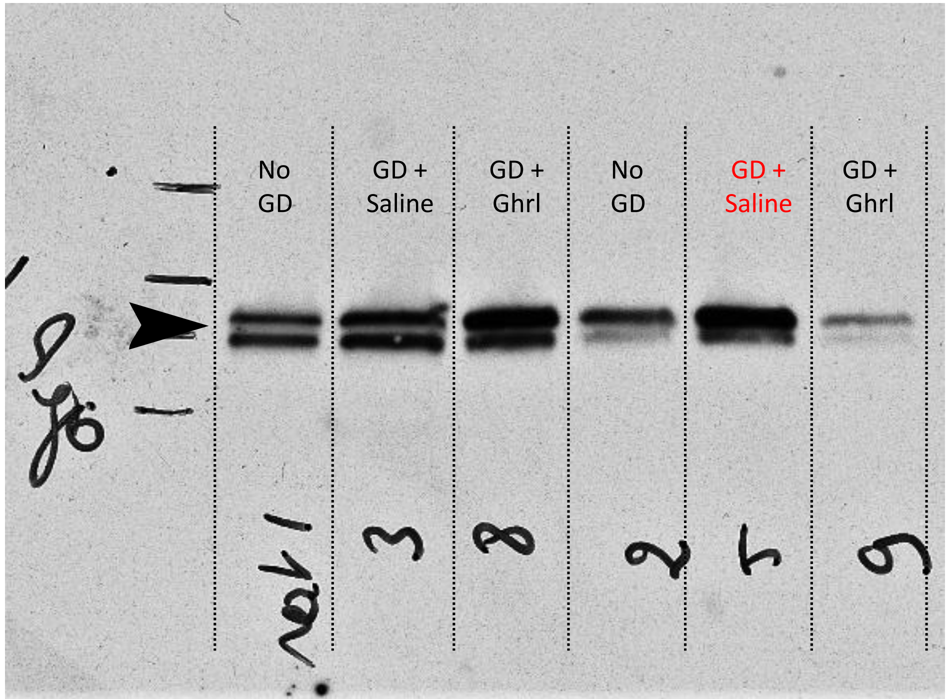

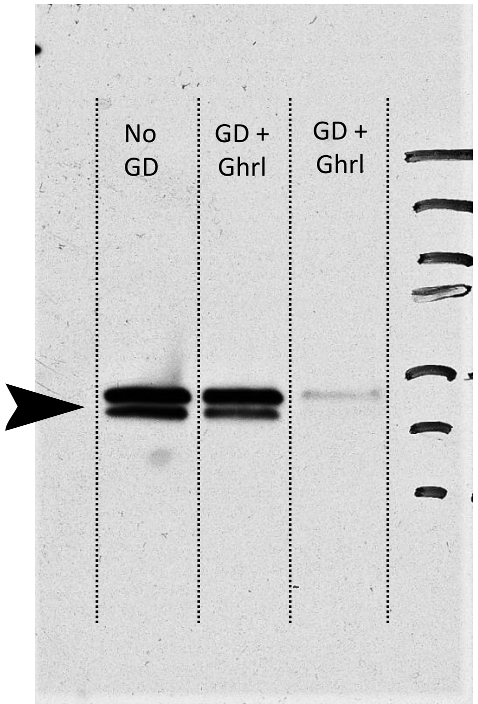

GD: Gastric distension
Ghrl: Ghrelin

Nodose Ganglia p-ERK

Experiment 2

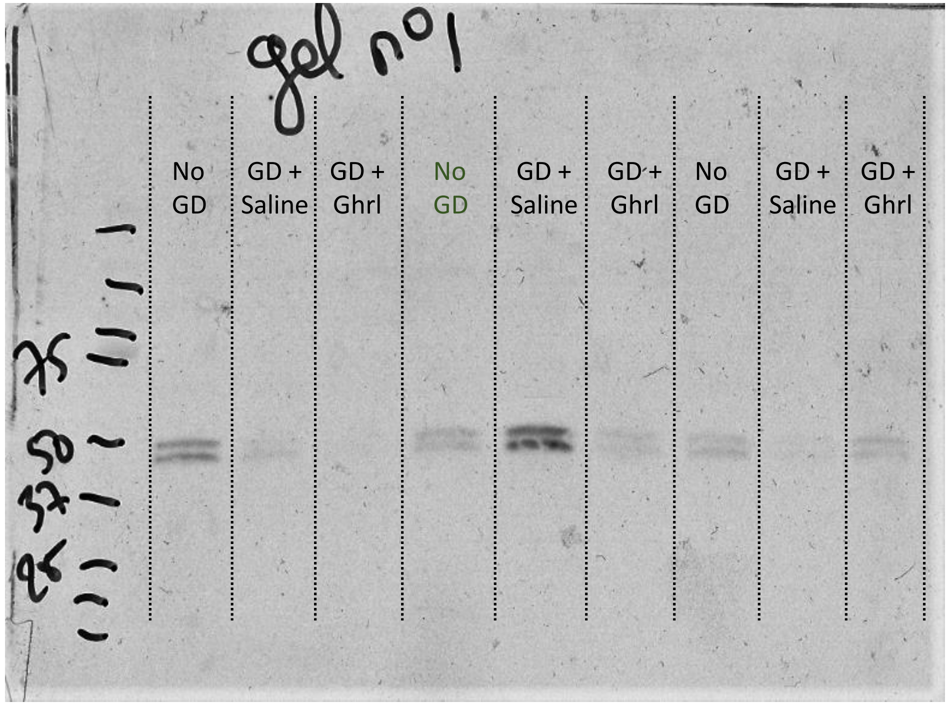

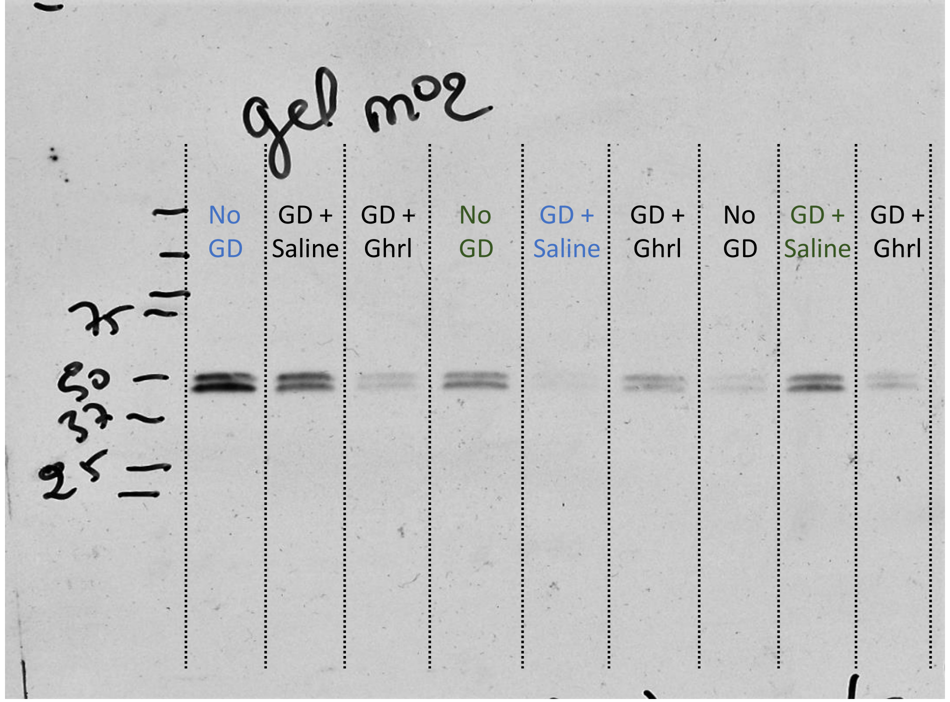

Data excluded because of animal death before the end of gastric stimulation

Outliers identified using the ROUT method (Q=1%) and excluded from analysis

Nodose Ganglia Total ERK

Experiment 2

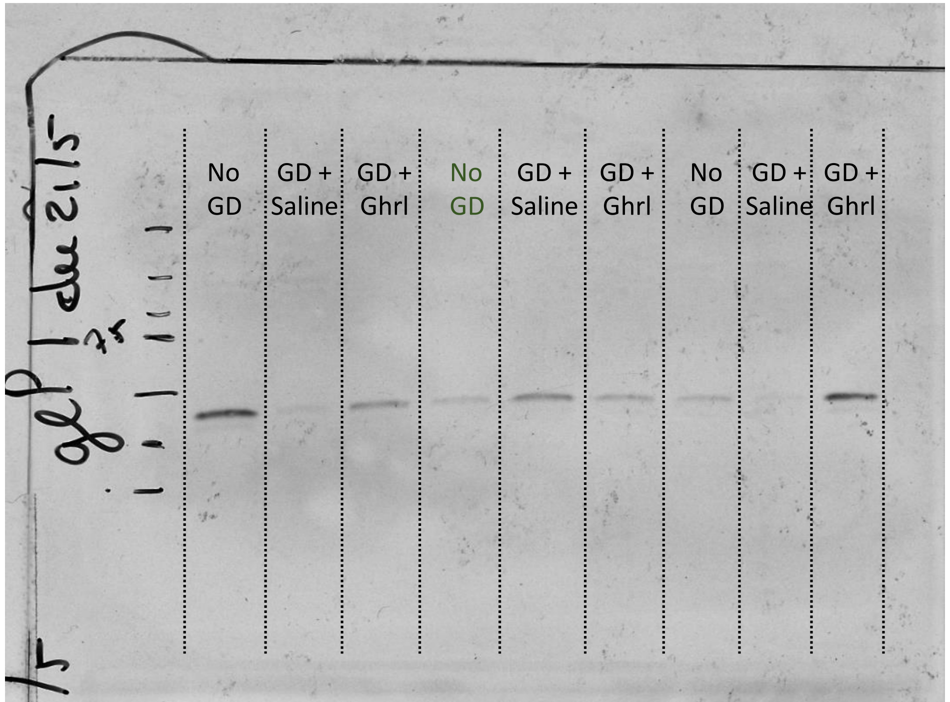

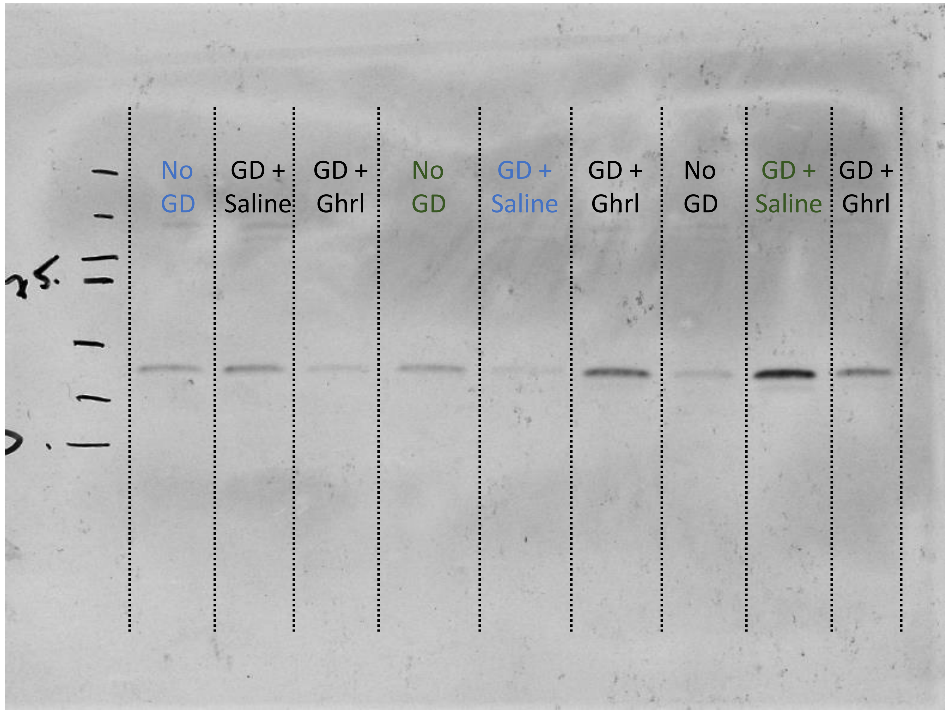

GD: Gastric distension

Ghrl: Ghrelin

Nodose Ganglia
p-ERK

Experiment 3

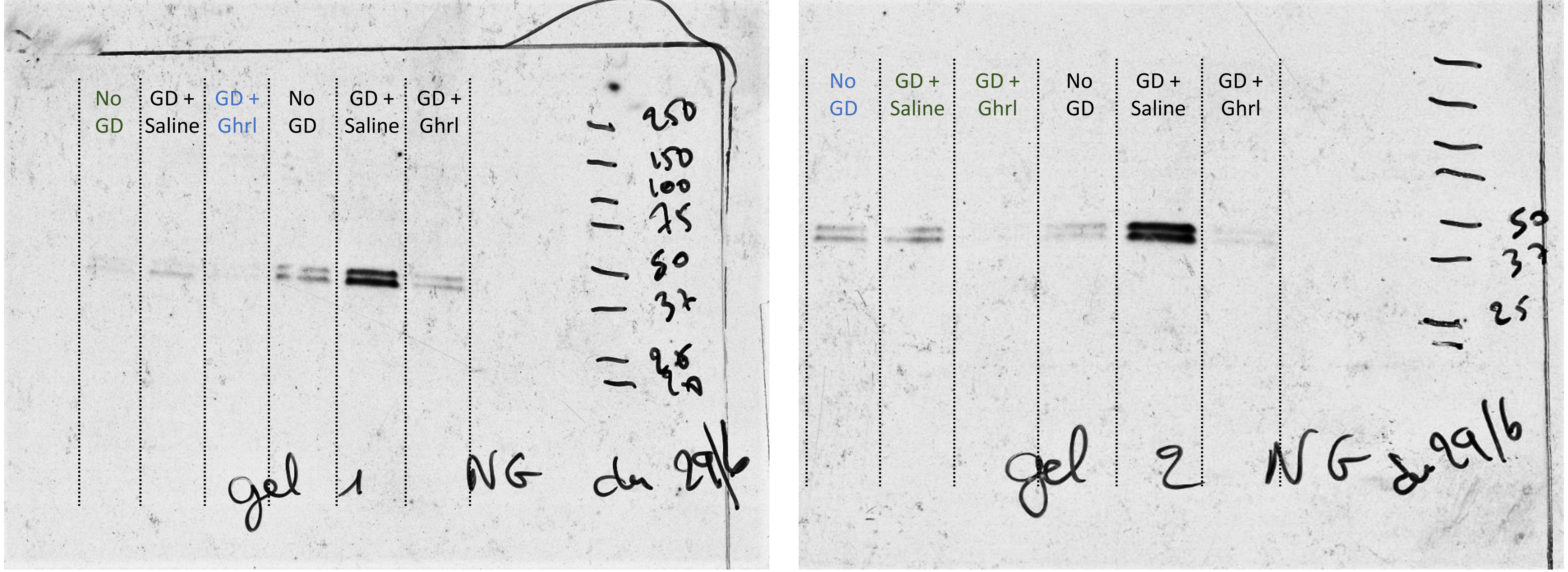

Nodose Ganglia
Total ERK

Experiment 3

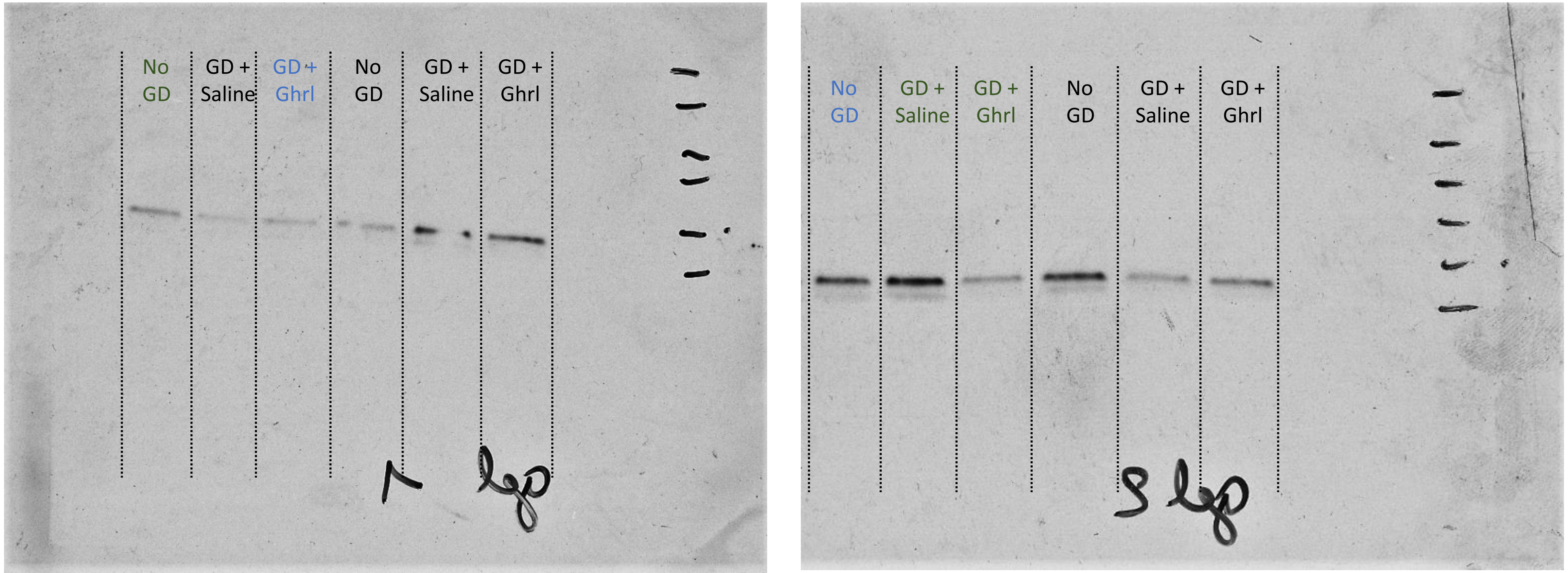

Data excluded because of animal death before the end of gastric stimulation

Outliers identified using the ROUT method (Q=1%) and excluded from analysis

GD: Gastric distension
Ghrl: Ghrelin

Dorsal Root Ganglia
p-ERK

Experiment 1

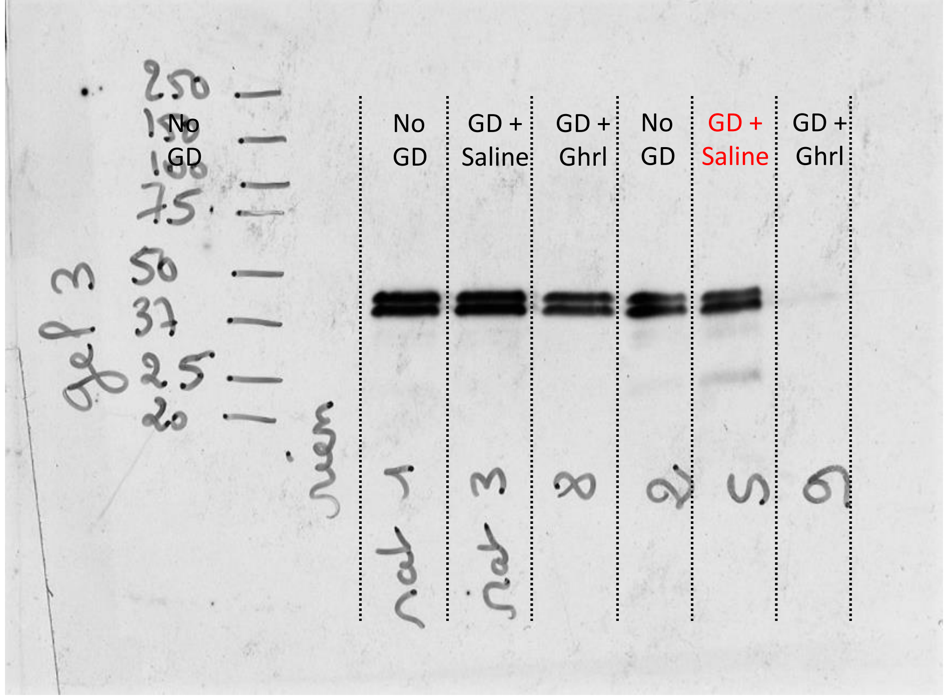

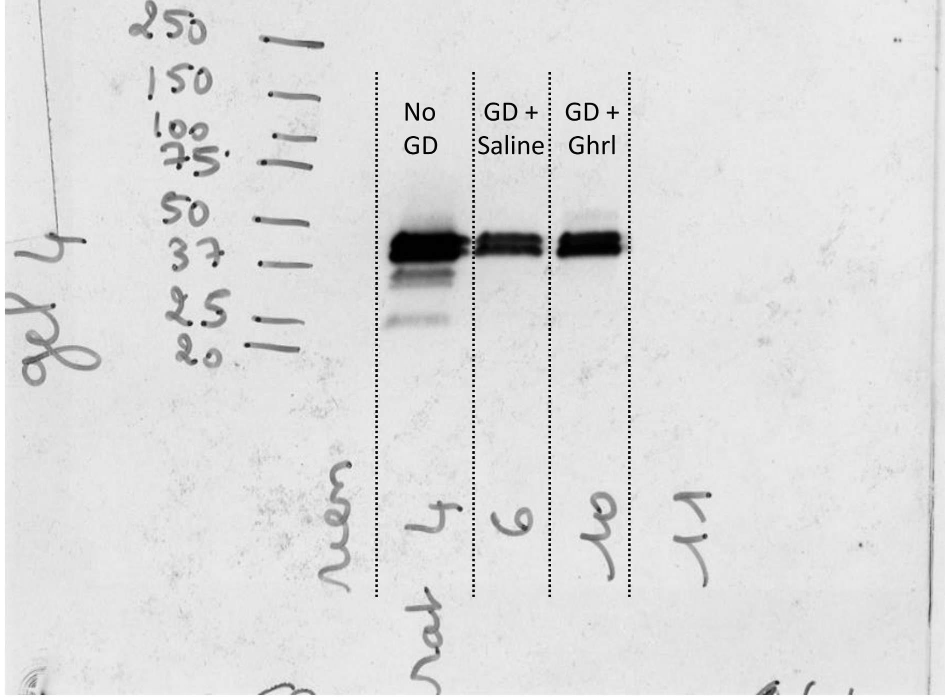

Data excluded because of a leak in gastric balloon to the small intestine

Dorsal Root Ganglia
Total ERK

Experiment 1

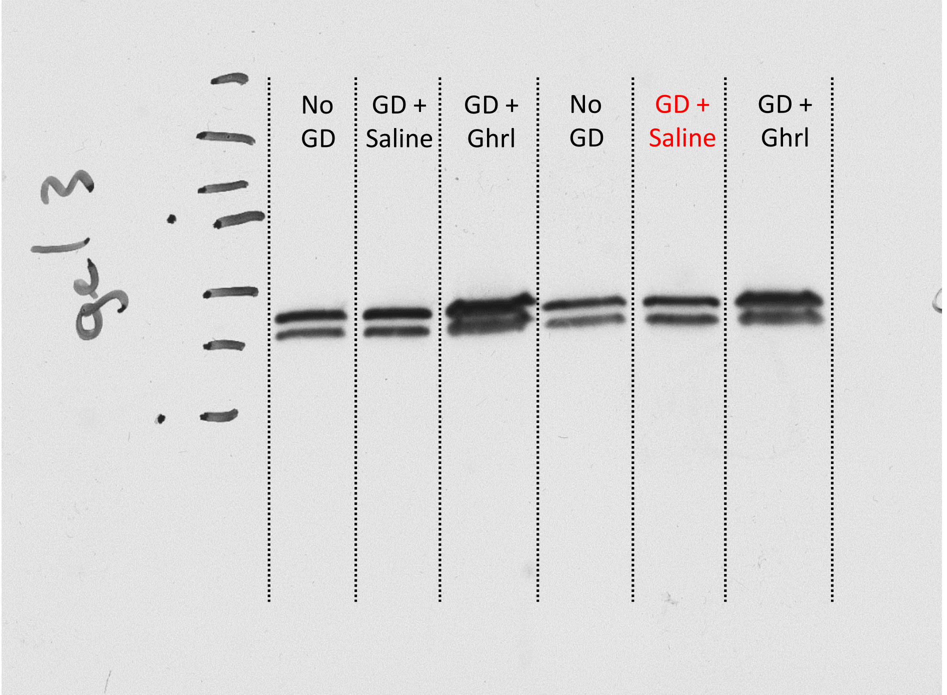

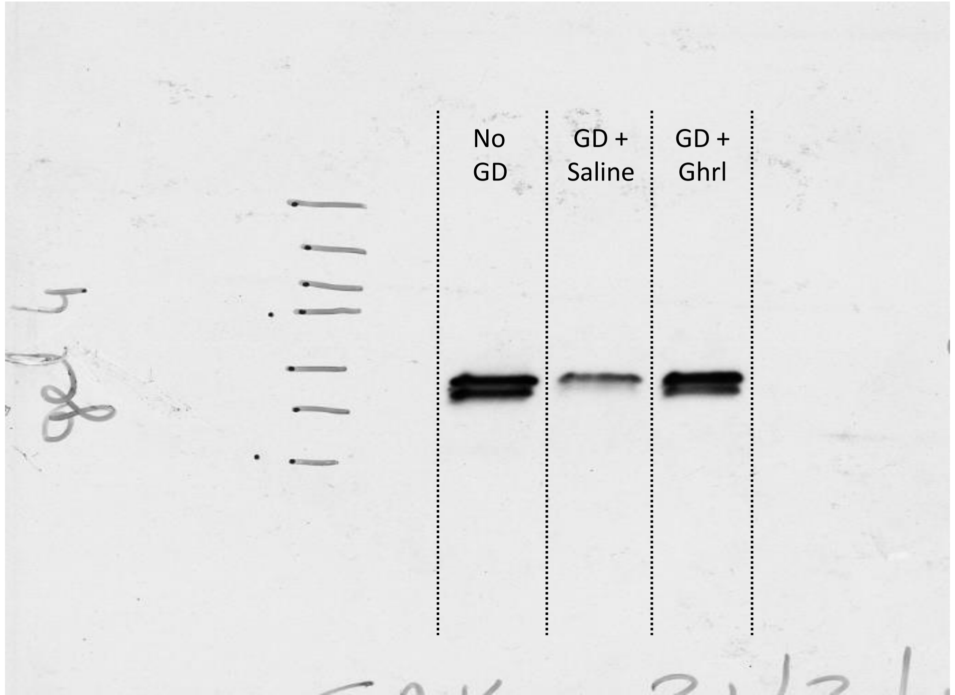

GD: Gastric distension
Ghrl: Ghrelin

Dorsal Root Ganglia p-ERK

Experiment 2

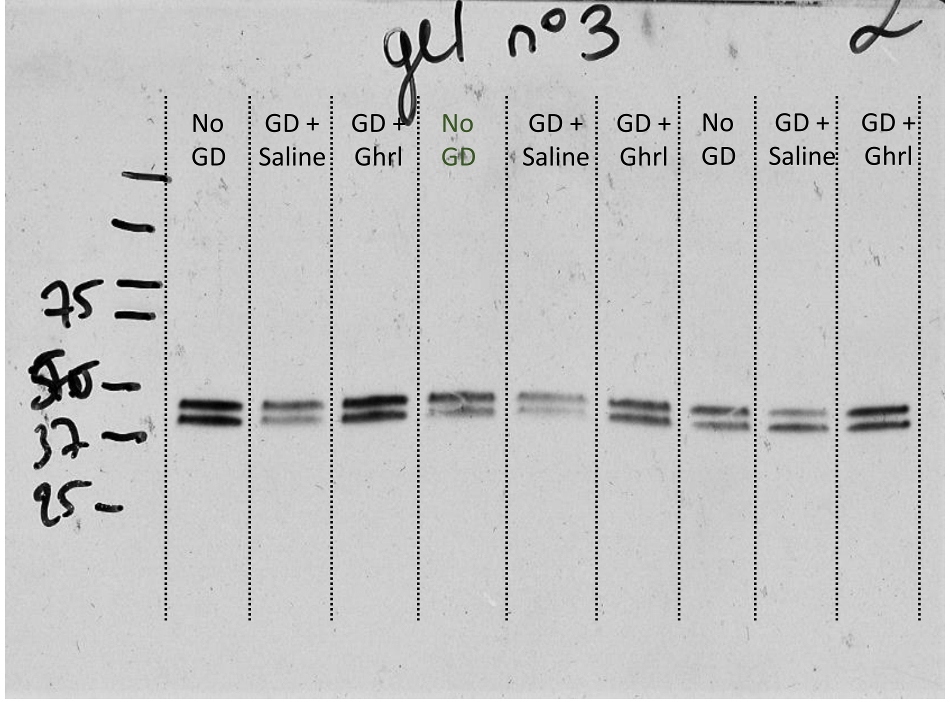

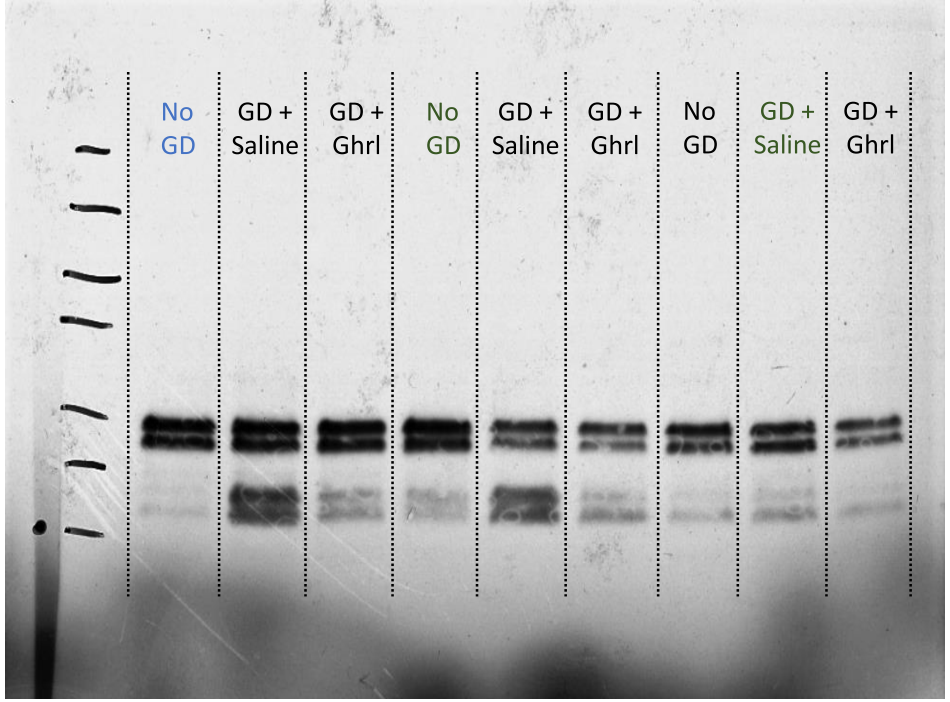

Data excluded because of animal death before the end of gastric stimulation

Outliers identified using the ROUT method (Q=1%) and excluded from analysis

Dorsal Root Ganglia Total ERK

Experiment 2

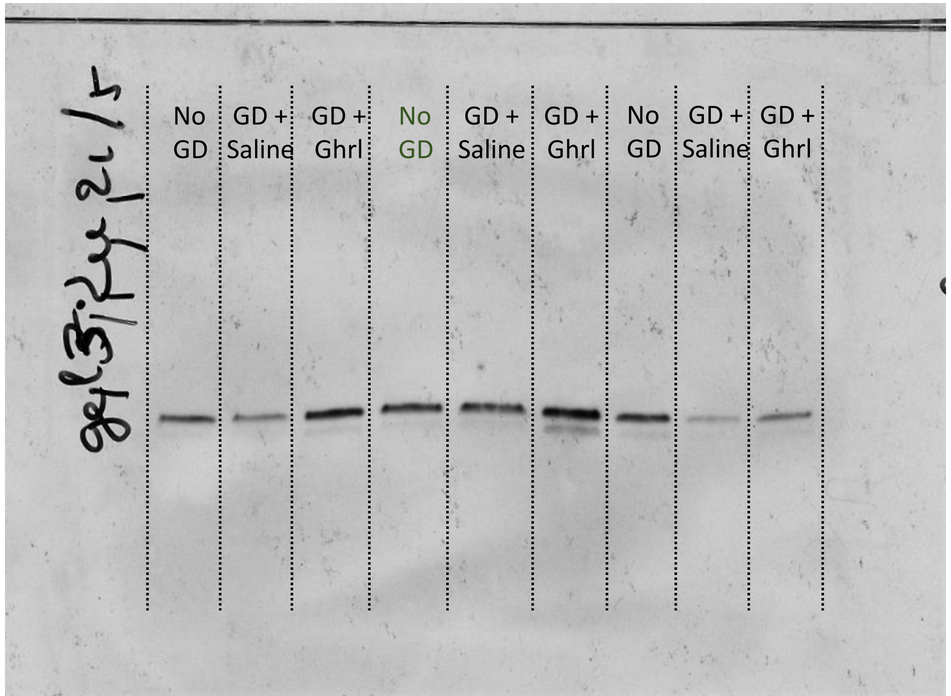

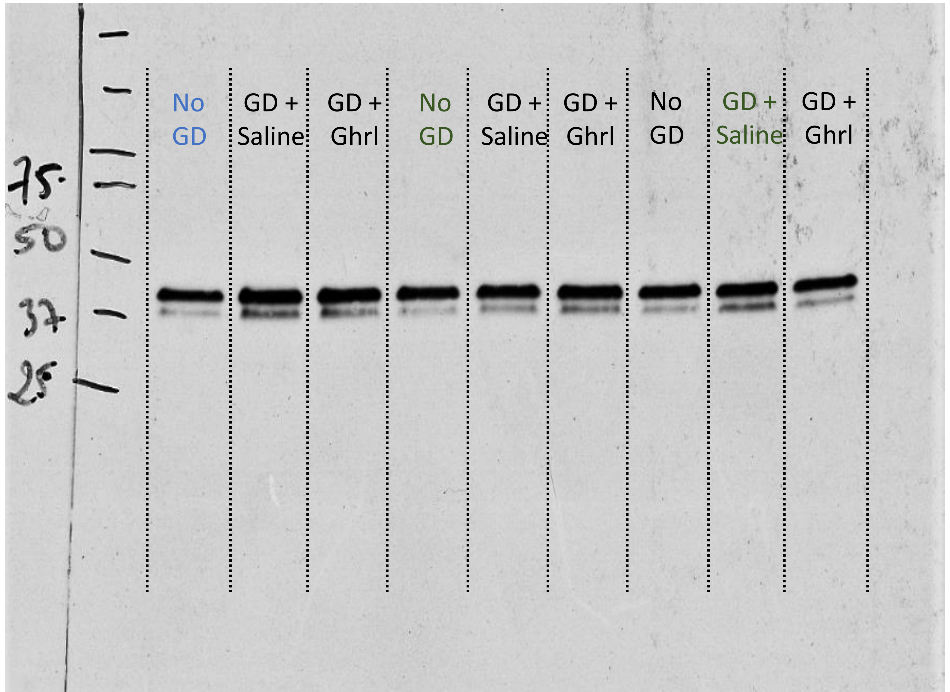

GD: Gastric distension
Ghrl: Ghrelin

Dorsal Root Ganglia p-ERK

Experiment 3

Dorsal Root Ganglia Total ERK

Experiment 3

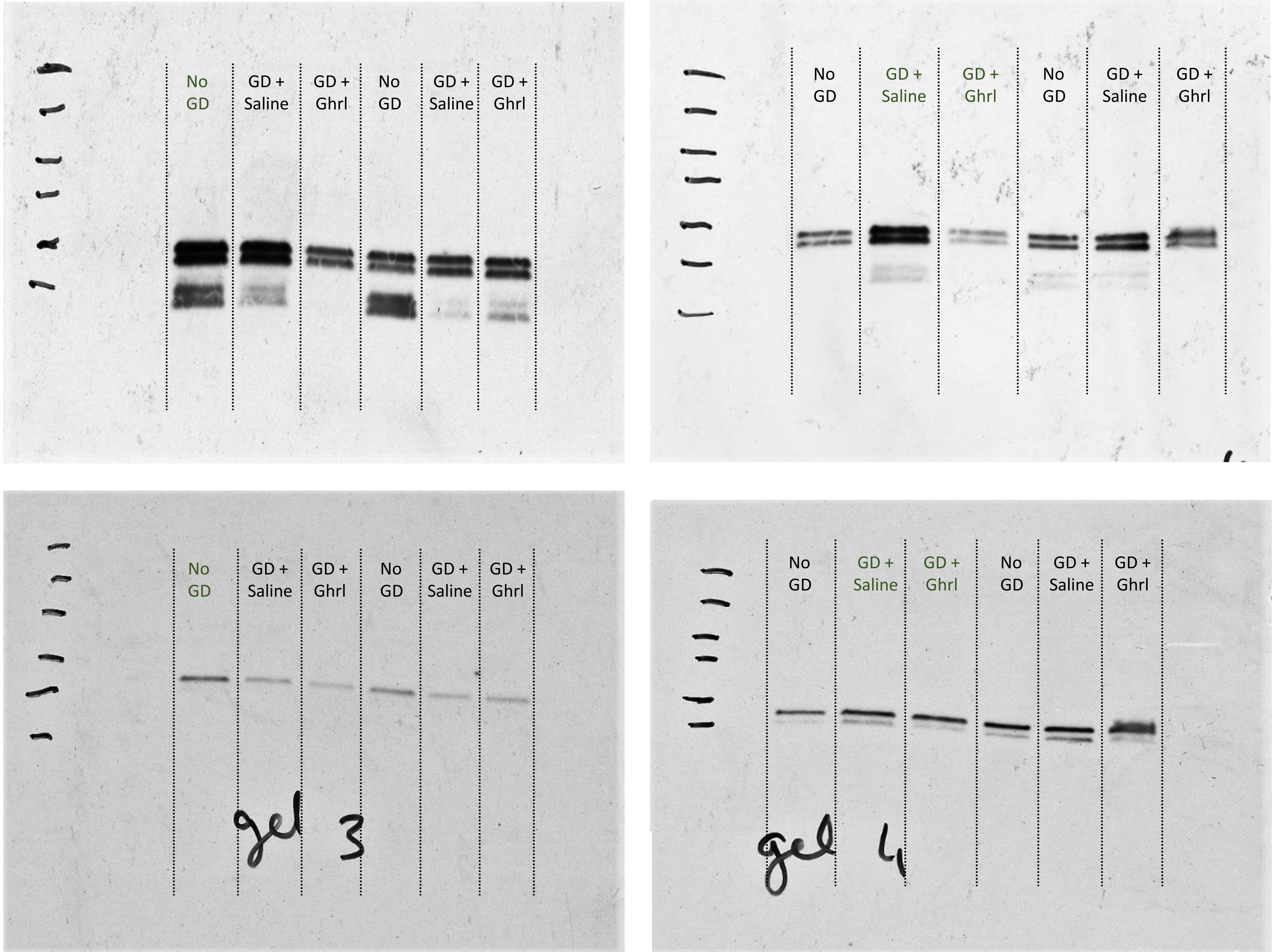

Data excluded because of animal death before the end of gastric stimulation

Outliers identified using the ROUT method (Q=1%) and excluded from analysis

GD: Gastric distension
Ghrl: Ghrelin
